# Usage of Hounsfield unit to differentiate idiopathic condylar resorption: a preliminary study

**DOI:** 10.22514/jofph.2026.005

**Published:** 2026-01-12

**Authors:** Kazuki Takata, Keiichiro Watanabe, Shinetsetseg Ser-od, Od Bayarsaikhan, Naoki Maeda, Susumu Abe, Eiji Tanaka

**Affiliations:** ^1^Department of Orthodontics and Dentofacial Orthopedics, Tokushima University Graduate School of Biomedical Sciences,770-8504 Tokushima, Japan; ^2^Department of Orthodontics, School of Dentistry, Mongolian National University of Medical Sciences, 15120 Ulaanbaatar, Mongolia; ^3^Department of Oral and Maxillofacial Radiology, Tokushima University Graduate School of Biomedical Sciences, 770-8504 Tokushima, Japan; ^4^Department of Comprehensive Dentistry, Tokushima University Graduate School of Biomedical Sciences, 770-8504 Tokushima, Japan

**Keywords:** Hounsfield unit, Computed tomography, Temporomandibular joint, Idiopathic condylar resorption

## Abstract

**Background**: The Hounsfield unit (HU) is a quantitative scale used to describe radiodensity in computed tomography (CT) scans. Since idiopathic condylar resorption (ICR) and temporomandibular joint osteoarthritis (TMJOA) involve destruction of bone and cartilage in the mandibular condyle, we hypothesized that HU values might be used to differentiate between the two conditions. This study aimed to evaluate the usefulness of HU values in the differential diagnosis of ICR and TMJOA. **Methods**: Twelve TMJOA and 9 ICR patients, and 11 healthy subjects were recruited as the TMJOA, ICR, and control groups, respectively. CT scans were performed, and HU values were measured in the region of interest (ROI) with 5 mm thickness along the Z-axis from superior condylar surfaces. HU distributions were then analyzed for each ROI. **Results**: Control and TMJOA patients were significantly older than those in the ICR group. Median HU values of the mandibular condyle did not differ significantly among the three groups. All groups showed a unimodal HU distribution peaking at 250–450 HU, while ICR condyles exhibited a tendency to have an additional peak at 1350–1500 HU. Compared to the control group, the HU distribution of the TMJOA and ICR condyles was significantly lower at 250–450 HU. After age adjustment, significant intergroup differences in the voxel ratio were noted at each HU level at 250–300, 300–350, 400–450, 1400–1450, and 1800–1850 HU. However, no significant differences in HU values were observed between the ICR and TMJOA groups. **Conclusions**: HU values and distributions of the mandibular condyle may be used to differentiate between the control group and the ICR and TMJOA groups. Further studies with a sufficient sample size are needed to confirm whether HU values and distribution could become important indicators for distinguishing between the TMJOA and ICR condyles.

## 1. Introduction

Idiopathic condylar resorption (ICR) is a rare disease affecting the temporomandibular joint (TMJ) and commonly occurs in teenage (around 12–18 years old) and postmenopausal (over 50 years old) females [1]. Patients with ICR present with rapid and severe condylar resorption, as well as a considerable reduction in mandibular ramus height. This leads to mandibular retrusion and clockwise rotation, resulting in an anterior open bite [2]. While the exact cause of ICR remains unknown, previous studies have identified several risk factors, such as age, sex, joint loading from orthodontic treatment, orthognathic surgery, trauma, postural and parafunctional habits, and internal derangement of the TMJ [1, 3, 4, 5]. Once the breakdown of the joint begins, ICR can lead to debilitating dentofacial deformities. Early identification and intervention to modify the skeleton are crucial to avoid severe skeletal deformities and irreversible damage to the temporomandibular joint (TMJ). Therefore, accurate and timely diagnosis of idiopathic condylar resorption (ICR) has become a matter of urgency [2].

Apart from ICR, temporomandibular disorders (TMDs) affect less than 10% of the population, with up to 31% of the elderly exhibiting TMD symptoms such as pain and sound [6, 7, 8]. Temporomandibular joint osteoarthritis (TMJOA) represents a degenerative disorder and stands as the most prevalent pathological condition affecting the joint. A survey on the epidemiology of the condition revealed minimal flattening of the condyle and/or eminence in 9.8% of elderly people [7]. Diagnosis of late-stage TMJOA is usually straightforward, particularly in cases of high inflammatory arthritic diseases. The problem arises in diagnosing the uncommon individuals whose arthritic condition is caused by ICR. Late-stage TMJOA and ICR condyles are both subject to significant bone resorption and severe bone deformity. It is critical to determine whether the condylar resorption is associated with TMJOA or ICR, in order to define the appropriate timing and management remedy.

The Hounsfield unit (HU) is a quantitative scale used to describe radiodensity. It is frequently used in computed tomography (CT) scans, where it is also referred to as the CT number. The HU values are derived by converting the original measurement of the attenuation coefficient into one where the radiodensity of distilled water at standard pressure and temperature is defined as zero [9, 10]. HU-based differentiation of materials is applicable to medical CT scans but not cone-beam CT scans, as the latter provide unreliable HU scale readings [11]. A recent study reported threshold values for classifying bone as normal or osteoporotic based on HU values, suggesting that HU values could be useful for classifying TMJOA and ICR [12].

The present study aimed to evaluate the effectiveness of HU values in determining whether condylar resorption was associated with TMJOA or ICR. The null hypothesis was that there would be no difference in HU values and distributions between the two conditions. The alternative hypothesis was that there might be differences in HU values and distributions between ICR and TMJOA, which could provide information to support earlier and more accurate differentiation. This could potentially improve diagnostic accuracy and reduce the need for surgical intervention.

## 2. Patients and methods

### 2.1 Participants

This retrospective cohort study recruited outpatients with TMD-associated symptoms who had visited the TMJ Clinic and/or the Orthodontic Clinic at Tokushima University Hospital between April 2014 and December 2024. Participants were excluded if they had a congenital craniofacial anomaly; traumatic injury to the TMJ region; rheumatic arthritis; systemic bone disease; unidentified symptoms of TMD; or orofacial pain. Each participant was evaluated for TMD symptoms and signs via clinical examination and a questionnaire, based on the diagnostic criteria for TMDs (DC/TMD) [13]. Moreover, if erosive changes and/or severe deformity of the mandibular condyle were evident in the initial panoramic radiograph, CT scans were performed using a medical CT machine (Aquilion, Toshiba Co., Tokyo, Japan). The final diagnosis for ICR and TMJOA was based on long-term follow-up and the repeated CT imaging (Fig. 1).

**Fig. 1.** Representative sagittal images of the TMJ. (A) Healthy condyle. (B) TMJOA condyle. (C) ICR condyle.

For the control group, subjects were recruited from a pool of healthy orthodontic patients whose chief complaints were malocclusion and esthetic problems. These patients were recruited from the Orthodontic Clinic at Tokushima University Hospital. The exclusion criteria were the same as for the TMJOA and ICR groups. Inclusion criteria were an absence of TMJOA and ICR, meaning healthy TMJs with adequate size and shape. Control patients were diagnosed with jaw deformity and/or a narrow maxillary arch, requiring orthognathic surgery and/or miniscrew-assisted rapid maxillary expansion. CT scans were performed for clinical use.

The study was approved by the Ethics Committee of Tokushima University Hospital (approval numbers 3050 since December 2017 and 2279 since April 2015). Informed consent was obtained from each patient after the research purposes and procedures had been fully explained.

### 2.2 CT examination

CT scans were performed using a multi-detector CT scanner (Aquilion, Toshiba Co., Tokyo, Japan) under the following conditions. The slice thickness was set to 1.0 mm and the field of view to 240.0 mm. This resulted in an in-plane resolution (the targeted voxel size) of 0.468 × 0.468 mm^2^. No additional beam filters were used in these scans. The imaging process yielded images with a pixel matrix size of 0.468 mm by 0.468 mm. The X-ray tube voltage was maintained at 120 kV and the tube current to 300 mA; the rotation duration was fixed at 0.5 s. Images were reconstructed using a bone algorithm reconstruction kernel. CT images were used to compare HU values and their distribution among the three groups. The region of interest (ROI) was determined by digitally cutting the mandibular condyle to a thickness of 5 mm along the Z-axis, starting from the superior surface of the condyle. This thickness was chosen to include the subchondral region where pathological changes predominantly occur, while avoiding the inclusion of deeper, non-pathological bone. The condyle regions were then manually isolated using ImageJ software (NIH). The distribution of the acquired HU values was assessed individually for each ROI. The HU value distribution was divided into 50 HU intervals, because a smaller interval would be hectic and a larger interval might make it harder to study the HU difference efficiently. The values were then compared and analyzed among the three groups (Fig. 2). HU measurements were performed by an experienced examiner. Although group allocation was blinded during the voxel extraction process, complete blinding was not possible due to morphological differences among the groups.

**Fig. 2.** Representative clinical CT image in the axial plane and a histogram of HU values. (A) Healthy condyle. (B) TMJOA condyle. (C) ICR condyle. Min: the minimum HU value; Max: the maximum HU value; StdDev: standard deviation. The yellow arrows indicate the segmented ROIs of the condyle extracted from the CT image (A).

### 2.3 Statistical analysis

Statistical analyses were executed with the aid of SPSS version 27.0 (SPSS Inc., Chicago, IL, USA) and R software version 4.5.0 (R Foundation for Statistical Computing, Vienna, Austria). Assessment of the normality of the data was undertaken using the Shapiro-Wilk test and evaluation of the box plot. Non-normally distributed data were expressed as the median with interquartile range and were analyzed. The difference in the sex ratio between the three groups was evaluated using Pearson’s chi-squared test. However, when the number of cells in the contingency table was less than five, the differences were evaluated using Fisher’s exact test. Differences in HU value intervals, total number of voxels within each ROI and median HU values of the mandibular condyle were evaluated among the ICR, TMJOA and control groups. A Kruskal-Wallis test was performed to compare the three groups for non-normally distributed data, including age, the number of voxels, and HU value intervals. If the result of the Kruskal-Wallis test was less than 0.050, intergroup comparisons were performed using an unpaired Mann-Whitney U test with Bonferroni correction. Otherwise, if the statistical results were found to be influenced by a confounding factor through a series of statistical processes was found, analysis of covariance (ANCOVA) was performed considering the covariates. Statistical significance was set at *p * < 0.050.

## 3. Results

A total of 9 patients suspected of ICR and 12 patients diagnosed with TMJOA were identified, while 11 individuals exhibiting minimal or no erosive changes alongside abnormal mandibular condyle deformities served as the control group. The male-to-female ratios were 1:8 (one male and eight females) in the ICR group, 1:3 (three males and nine females) in the TMJOA group, and 3:8 (three males and eight females) in the control group (Fisher’s exact test, *p* = 0.648). The average age at the initial visit was 16 years (range 13.5–30.0 years), 45.5 years (range 32.8–63.0 years), and 35.0 years (range 25.0–46.0 years) in the ICR, TMJOA, and control groups, respectively. ICR patients were significantly younger than the TMJOA and control groups (*p* = 0.005). The sample size was determined by the number of eligible patients who underwent a CT scan at Tokushima University Hospital during the study period and met the inclusion and exclusion criteria. Although no priori sample size calculation was performed, all qualifying cases were included, resulting in nine patients with ICR, 12 patients with TMJOA, and 11 control subjects. This number represented the maximum sample attainable under the study conditions.

The total number of voxels within each ROI was 1559 (1373–1799) in the ICR group,1789 (1550–1906) in the TMJOA group, and 1837 (1653–2233) in the control group. A significant difference in the total number of voxels was found between the control and ICR groups; the control group exhibited a significantly higher number of voxels (*p* = 0.023). No statistically significant differences were observed between the ICR and TMJOA groups (*p* = 0.390) or between the TMJOA and control groups (*p* = 0.604). In terms of HU distribution, all three groups exhibited a unimodal distribution based on the proportion of voxels relative to the total number of voxels within each ROI, peaking at 250–450 HU (Fig. 3A). In contrast, ICR condyles showed a tendency to have an additional peak at 1350–1500 HU (Fig. 3B). CT measurements revealed median HU values (interquartile range) of 606.3 (453.8–745.5) for the mandibular condyle in the ICR group, 586.1 (456.34–692.7) for the TMJOA group, and 515.3 (409.4–626.3) for the control group. No significant differences in the median HU values were found among the three groups (*p* = 0.347). Table 1 shows the differences in voxel ratio between groups at each HU level. TMJOA condyles exhibited significantly less distribution at 200–250 HU (*p* = 0.022), 250–300 HU (*p* = 0.003), 300–350 HU (*p* = 0.004), and 400–450 HU (*p* = 0.011), as well as significantly more distribution at 900–950 HU (*p* = 0.045), 1150–1200 HU (*p* = 0.037), 1200–1250 HU (*p* = 0.047), 1250–1300 HU (*p* = 0.041), 1350–1400 HU (*p* = 0.027), 1400–1450 HU (*p* = 0.004), 1450–1500 HU (*p* = 0.019), 1500–1550 HU (*p* = 0.003) and 1550–1600 HU (*p* = 0.033) compared to control condyles. The condyles affected by ICR showed a significantly decreased distribution within the HU ranges of 200–250 (*p* = 0.021), 250–300 (*p* = 0.038) and 300–350 (*p* = 0.020), alongside a significantly increased distribution within the ranges of 1400–1450 HU (*p* = 0.010) and 1500–1550 HU (*p* = 0.029), compared to control condyles. However, no significant differences were found between the ICR and TMJOA groups, regardless of the HU values. To account for the potential confounding effect of age in the comparison among the three groups, nonparametric rank-based permutation ANCOVA was applied to evaluate the overall distributional pattern of HU values. Age was found to have a significant effect (*p* = 0.002), indicating its role as a confounding factor. Furthermore, group effects were also significant (*p* = 0.002), suggesting that statistically significant differences existed among the three groups even after adjusting for age. Next, to investigate regional differences, each of the HU value region was tested separately using rank ANCOVA with age as a covariate. Table 2 shows the intergroup differences in the voxel ratio at each HU level at 250–300 HU (*p* = 0.019), 300–350 HU (*p* = 0.019), 400–450 HU (*p* = 0.046), 1400–1450 HU (*p* = 0.019) and 1800–1850 HU (*p* = 0.044). These five regions showed significant group effects after false discovery rate (FDR) correction using the Benjamini–Hochberg method. Furthermore, for the five significant regions, age-adjusted residuals were obtained from the rank-based ANCOVA as the *post-hoc* test. These *post-hoc* tests with FDR correction revealed significant differences between the control group and the ICR and TMJOA groups in all five regions (Table 2). However, comparisons between the ICR and TMJOA groups were generally not significant.

**Fig. 3.** Histogram illustrating the distribution of HU values on the surface of mandibular condyles. Blue, Control group; Orange, ICR group; Gray, TMJOA group. The voxel ratio at each HU value was calculated by dividing the number of voxels in each HU bin by the total number of voxels within the segmented region. The control group showed higher voxel ratios at approximately 200–400 HU, and lower ratios around 1400 HU, compared to the TMJOA and ICR groups. Region (A) (highlighted in green) represents a primary peak common to all groups. Region (B) (highlighted in blue) shows a secondary peak observed exclusively in the ICR group. HU: Hounsfield unit; TMJOA: temporomandibular joint osteoarthritis; ICR: idiopathic condylar resorption.

**Table 1.** Intergroup differences in voxel ratio for each HU value.

HU Value	Control (×10^−2^)	ICR (×10^−2^)	TMJOA (×10^−2^)	*p*-Value	*Post-hoc* test		
Range	Median (25%–75%)	Median (25%–75%)	Median (25%–75%)		Control-ICR	ICR-TMJOA	TMJOA-Control
200–250	6.09 (4.72–7.17)	4.41 (2.82–5.22)	5.07 (4.31–5.54)	**0.022**	**0.021**	1.000	0.179
250–300	6.65 (5.70–9.00)	5.16 (3.01–5.91)	4.31 (2.99–5.66)	**0.003**	**0.038**	1.000	**0.003**
300–350	6.91 (5.24–9.68)	4.08 (3.11–5.60)	4.39 (3.79–5.28)	**0.004**	**0.020**	1.000	**0.009**
400–450	6.85 (5.23–7.82)	4.47 (2.90–5.40)	4.05 (3.50–4.78)	**0.011**	0.064	1.000	**0.015**
900–950	1.32 (1.02–2.71)	2.30 (1.83–2.71)	2.82 (2.08–3.24)	**0.045**	1.000	0.518	**0.040**
1150–1200	1.27 (0.51–1.91)	2.31 (1.24–2.56)	1.97 (1.52–2.53)	**0.037**	0.079	1.000	0.083
1200–1250	1.18 (0.51–1.41)	1.99 (1.08–3.00)	1.89 (1.15–3.47)	**0.047**	0.291	1.000	**0.049**
1250–1300	0.93 (0.39–1.36)	1.31 (0.81–2.84)	1.87 (1.04–3.00)	**0.041**	0.438	1.000	**0.036**
1350–1400	0.77 (0.44–0.98)	1.65 (0.74–3.10)	1.49 (0.89–1.99)	**0.027**	0.058	1.000	0.066
1400–1450	0.54 (0.27–1.06)	1.57 (0.85–3.61)	1.47 (0.78–2.06)	**0.004**	**0.010**	1.000	**0.020**
1450–1500	0.44 (0.27–1.05)	1.21 (0.71–3.19)	1.07 (0.83–1.53)	**0.019**	0.056	1.000	**0.041**
1500–1550	0.43 (0.24–0.80)	1.07 (0.74–4.19)	1.22 (0.90–1.56)	**0.003**	**0.029**	1.000	**0.004**
1550–1600	0.42 (0.19–0.78)	0.86 (0.47–3.22)	1.06 (0.67–1.60)	**0.033**	0.143	1.000	**0.043**

*Kruskal-Wallis test was used followed by pairwise comparison by multiple Mann Whitney (significance level *p * < 0.050).* **p*-values are Bonferroni corrected for multiple pairwise comparisons.* *The bold numbers in the table indicate values with *p * < 0.050.* *HU: Hounsfield unit; ICR: idiopathic condylar resorption; TMJOA: temporomandibular joint osteoarthritis.*

**Table 2.** Intergroup differences in voxel ratio for each HU value with age as a covariate.

HU Value	Control (×10^−2^)	ICR (×10^−2^)	TMJOA (×10^−2^)	*p*-Value	*Post-hoc* test		
Range	Median (25%–75%)	Median (25%–75%)	Median (25%–75%)		Control-ICR	ICR-TMJOA	TMJOA-Control
250–300	6.65 (5.70–9.00)	5.16 (3.01–5.91)	4.31 (2.99–5.66)	**0.019**	**0.008**	0.862	**0.004**
300–350	6.91 (5.24–9.68)	4.08 (3.11–5.60)	4.39 (3.79–5.28)	**0.019**	**0.006**	0.508	**0.003**
400–450	6.85 (5.23–7.82)	4.47 (2.90–5.40)	4.05 (3.50–4.78)	**0.046**	0.015	0.808	**0.008**
1400–1450	0.54 (0.27–1.06)	1.57 (0.85–3.61)	1.47 (0.78–2.06)	**0.019**	**0.010**	1.000	**0.020**
1800–1850	0.40 (0.00–0.54)	0.21 (0.00–0.44)	0.26 (0.09–0.47)	**0.044**	0.766	0.122	**0.002**

*The rank ANCOVA test showed a significant difference between the HU values in the table.* **p*-values are false discovery rate (FDR) corrected using Benjamini-Hochberg method.* *The bold numbers in the table indicate values with *p * < 0.050.* *HU: Hounsfield unit; ICR: idiopathic condylar resorption; TMJOA: temporomandibular joint osteoarthritis.*

## 4. Discussion

Identifying the activity status of condylar resorption—whether active or inactive—is essential for selecting the optimal management approach and timing. Hatcher [14] proposed two methods for accessing stability in patients with progressive condylar resorption (PCR). One of these is nuclear medicine scanning, which provides immediate results. This approach generally entails the utilization of a bone scanning technique, such as a conventional bone scan employing technetium-99m methylene diphosphonate (^99m^Tc-MDP), or a Tc-MDP single-photon emission CT scan [15, 16]. However, despite the usefulness of bone scans for the evaluation of certain medical conditions, there may be insufficient specificity for the determination of stability (inactivity) in cases of condylar resorption [17]. Another approach includes reassessing and comparing condylar morphology at designated intervals. Time is the most useful tool for determining TMJ bony stability in PCR [14, 18]. It is recommended that, once the radiographic features of advanced condylar resorption have been obtained via CT, stability in the TMJ should be reevaluated via radiography 6–12 months later [14]. Nevertheless, the observation period following the remission stage may be excessively long, and there is no assurance that the resorptive process will not recommence once the selected management process is reinitiated.

In the present study, the HU values, and distributions on the surface of mandibular condyle were evaluated and compared between ICR and TMJOA patients. The median HU values of the mandibular condylar surfaces were found to be 606.3 (IQR (Interquartile Range), 453.8–745.5) in the ICR patients, 586.1 (IQR, 456.34–692.7) in TMJOA patients, and 515.3 (IQR, 409.4–626.3). The median HU value in TMJOA patients was nearly consistent with that in the ICR patients, and there were no significant differences in the median HU values among the three groups. Regarding HU distribution on the surface of the mandibular condyle, all three groups exhibited a unimodal distribution, peaking at 250–450 HU, while ICR condyles showed a tendency to have an additional peak at 1350–1500 HU. Compared to the control group, the HU distribution of the TMJOA and ICR condyles was significantly lower at 250–450 HU. One possible reason for the lower HU values being widely distributed on the surface of the ICR condyle is that the rapid bone resorption in the ICR makes it difficult for the surrounding bone defense mechanism, as seen in inflammatory bone resorption, to function. However, as the ICR patients were significantly younger than the control and TMJOA groups, the two peak distributions of the HU values may be associated with bone growth and remodeling. Then, to evaluate the differences in the voxel ratio among the three groups, we performed a rank-based ANCOVA adjusting for the possibility of confounding factors due to age differences. The results revealed significant differences in the voxel ratio between the groups, even after adjusting for age (*p* = 0.002). This indicates that HU distribution could be an effective way to identify the healthy condyle and the TMJOA and ICR condyles. Meanwhile, comparisons of HU values and distributions between the ICR and TMJOA groups were generally non-significant. Thus far, further studies with a sufficient sample size are needed to confirm whether HU values and distribution could become important indicators for distinguishing between the TMJOA and ICR condyles.

It has been demonstrated by earlier research that CT findings indicate marked variations in condylar dimensions and morphology in individuals with ICR as opposed to those with TMJOA [19, 20]. ICR development may be indicated by changes in condylar shape, as well as decreased width and height of the condyle. A decreased condylar axial angle may be a characteristic specific to ICR. Additionally, greater compressive stress is experienced by the lateral and anterior areas of the condyle during mouth opening [21]. This suggests that the process of bone remodeling in the lateral and medial regions of the condyle may differ during ICR development, thereby reducing the condylar axial angle. This is because the condylar head rotates superolaterally upon emergence from the ramus. This means that the condylar axial angle decreases with the progression of condylar resorption, and that the differences in condylar size and shape may be detectable in the late stage of ICR, but not in the early stage. The early detection of ICR may be facilitated by CT evaluation through HU distribution due to the potential for changes in HU values and distributions on the condylar surface to precede structural changes in the mandibular condyle.

In the subchondral bone, the microenvironment may reveal the presence of osteoclastic bone resorption activity in the early stages of OA, which can be distinguished from the late stages by an increase in bone formation [22]. Numerous vessels originating from the subchondral bone penetrate the calcified cartilage and infiltrate the non-calcified cartilage in OA joints [23]. Courtois and Ohnmeiss [12] investigated threshold values for classifying bone as normal or osteoporotic based on HU values. They concluded that HU values of >170 were indicative of normal bone, while HU values of <115 were indicative of osteoporosis. Overall, HU values may serve well as a new reference for classifying TMJOA and ICR.

This study has several limitations. Firstly, the small number of patients was due to the rarity of ICR, meaning that statistical adjustments for potential confounders such as age and sex could not be made without reducing the study’s statistical power. ICR patients were significantly younger and predominantly female compared with TMJOA patients; these demographic differences may therefore have contributed to the observed HU distributions. Furthermore, age and group factors together explained approximately 36% of the total variance, whereas the remaining 63.8% was attributed to residual variance. This relatively large unexplained component indicates that unmeasured confounding factors, individual differences, or potential measurement errors may have influenced the results. Therefore, future studies should aim to control for these unexplained sources of variance more comprehensively as well as involving a larger sample size, which is needed to evaluate the effectiveness of the HU values and distributions in ICR and TMJOA condyles. Secondly, as most clinical facilities rely on cone-beam CT rather than medical CT for TMJ imaging, our findings may be difficult to apply directly to routine practice. Future multicenter studies involving larger, age-matched cohorts are necessary to validate the present results.

## 5. Conclusions

The present study attempted to explore a new indicator for classifying ICR and TMJOA. Regarding HU distribution on the surface of the mandibular condyle, TMJOA and ICR condyles exhibited a unimodal distribution, peaking at 250–450 HU, as well as healthy condyles, while ICR condyles showed a tendency to have an additional peak at 1350–1500 HU. Furthermore, in the five specific HU levels, significant differences in the voxel ratio were found between the control and both patient groups (ICR and TMJOA), whereas comparisons between the ICR and TMJOA groups were generally non-significant. More studies with a sufficient sample size are required to determine the efficiency of HU to differentially diagnose ICR.
